# Methylation statuses of *NCOR2*, *PARK2*, and *ZSCAN12* signify densities of tumor-infiltrating lymphocytes in gastric carcinoma

**DOI:** 10.1038/s41598-022-04797-9

**Published:** 2022-01-17

**Authors:** Xianyu Wen, Hye-Yeong Jin, Meihui Li, Younghoon Kim, Nam-Yun Cho, Yoonjin Kwak, Jeong Mo Bae, Hye Seung Lee, Gyeong Hoon Kang

**Affiliations:** 1 Department of Pathology, Seoul National University College of Medicine, 103 Daehak-ro, Jongno-gu, Seoul, 03080 Korea; 2 Laboratory of Epigenetics, Cancer Research Institute, Seoul National University College of Medicine, Seoul, Korea

**Keywords:** Gastroenterology, Molecular medicine, Oncology

## Abstract

Individual cell types of human tissues have their own CpG site methylation profiles, which might be utilized for the development of methylation markers to denote tumor-infiltrating lymphocytes (TILs). We aimed to develop DNA methylation markers that recapitulate the densities of TILs in gastric carcinoma (GC). Through genome-wide methylation profiling, *NCOR2*, *PARK2*, and *ZSCAN12* were found to be highly methylated in CD3-positive and CD8-positive cells and rarely methylated in tumor cells. Scores of the three methylation markers were analyzed for their relationship with the overall survival and recurrence-free survival of patients with advanced GC (n = 471). The scores of three methylation markers were closely associated with densities of CD3-positive or CD8-positive cells at the tumor center or invasive front of GCs and found to be a significant prognostic factor in univariate analysis of overall survival and recurrence-free survival. In multivariate analysis, the highest score showed hazard ratios of 0.513 (CI 0.306–0.857) and 0.434 (CI 0.261–0.720) for overall survival and recurrence-free survival, respectively. The findings suggest that methylation markers signifying TILs might be utilized for the recapitulation of TIL density in GCs and serve as biomarkers for predicting prognosis in patients with GC.

## Introduction

Tumor-infiltrating lymphocytes (TILs) are mononuclear and polymorphonuclear immune cells that have left the bloodstream and migrated toward a tumor. An expanding body of evidence indicates that the extent and composition of TILs in the tumor microenvironment has prognostic significance in solid malignancies^1^. For gastric carcinoma (GC), the amount of TILs has been demonstrated to play an important role in predicting the clinical outcome of patients^2,3^. Moreover, assessment of TILs is recognized as an important predictive tool for selecting patients who are most likely to respond to immunotherapy drugs. Despite the importance of TIL assessment, the inclusion of TIL assessment in routine pathological reporting is hindered by the lack of standardized methodology for TIL assessment^4,5^. Recently, a pragmatic methodology for assessment of TILs on hematoxylin and eosin-stained sections was proposed by International Immuno-Onoclogy Biomarker Working Group^5^, and there are international efforts to validate its clinical utility regarding patient prognosis or response to treatment. In addition, the immunoscore, which was developed for TIL assessment in colorectal carcinoma by the Galon team, might have the potential to serve as an alternative tool for TIL assessment in GC^6^.

Cytosine methylation in which a methyl group is added to the cytosine of a CpG site in genomic DNA is important for maintaining cellular diversity in multicellular organisms. It has been demonstrated that various human tissues have their own characteristic methylation profiles, namely, tissue-specific methylation profiles^7,8^. Furthermore, each blood leukocyte cell type has been found to carry its own methylation profile^9^. Such a finding suggests that cell type-specific methylation patterns exist for TILs and that specific methylation markers can be utilized for assessment of TIL density. Recently, the Fuks team identified the methylation signature of breast cancer TILs and reported the value of DNA methylation in evaluating host immune responses to tumors^10^.

In the present study, we sought to develop DNA methylation markers whose methylation levels were correlated with the density of TILs in GC and to determine whether the methylation status of TIL-specific methylation markers is associated with the prognosis of patients with GC. By using a genome-wide methylation assay for CD3-positive and CD8-positive (CD3+/CD8+) cells versus CD3-negative and CD8-negative (CD3−/CD8−) cells, we developed three DNA methylation markers with methylation levels correlating with TIL density in GC. These methylation markers exhibit close associations with GC prognosis, and the sum of methylated markers is also independently associated with GC prognosis.

## Results

### Selection of DNA methylation markers that show correlation between their methylation levels and DNA concentration from tumor-infiltrating lymphocytes

**Figure 1:**
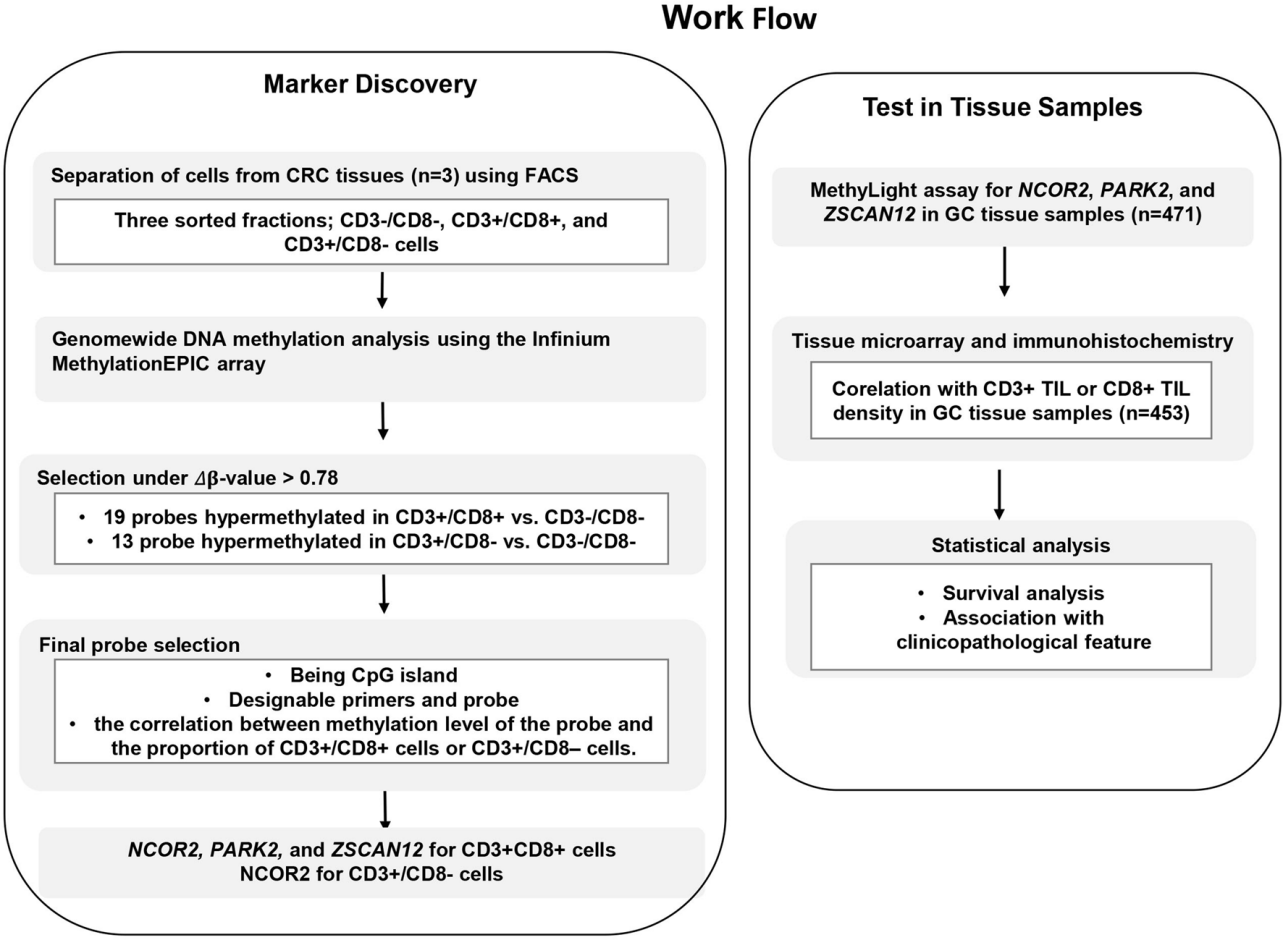
Overview of study workflow from marker discovery to verification in tissue samples. The process of selection of candidate DNA methylation markers and their verification in detection of CD8-positive T cells in tissue samples from patients with GC and their correlation with survival and clinicopathological features were schematically represented.

**Figure 2:**
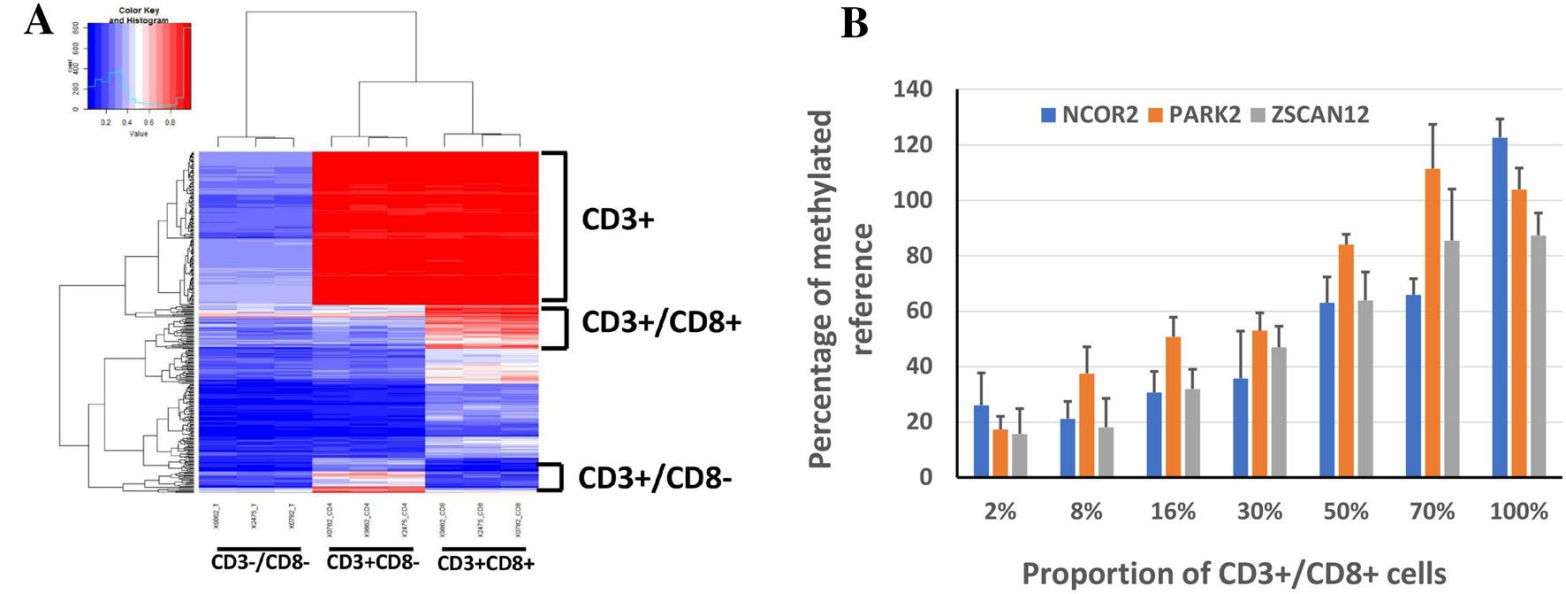
(**A**) CpG sites showing differences in methylation values in the three subsets (CD3−/CD8− subset, CD3+ CD8− subset, and CD3+ CD8+ subset) were 272,302 CpG sites. In the case of CD3+ subsets (CD3+/CD8− and CD3+/CD8+ subsets); 271,708 CpG sites were differentially methylated, of which 194,770 were hypermethylated. On the other hand, 307 CpG sites showed differences in methylation for the CD3+/CD8+ subset, of which 150 were hypermethylated. (**B**) The MethyLight assay was performed to identify whether the PMR values of *NCOR2*, *PARK2*, and *ZSCAN12* increase proportionally when the abundance of the CD3+/CD8+ subset among the total amount of CD3+/CD8+ subset and CD3−/CD8− subset is gradually increased. Spearman’s rho and *P*-value, 0.908 and < 0.001 for *NCOR2*; 0.916 and < 0.001 for *PARK2*; 0.566 and 0.007 for *ZSCAN12.*

The outline of the study is depicted in Fig. 1. Genomic DNA samples extracted from three subsets (CD3+/CD8+, CD3+/CD8−, and CD3−/CD8−) were subjected to the MethylationEPIC assay, which analyzes 870,000 CpG sites distributed across the human genome. Genome-wide methylation profiles between the three subsets were compared to find differentially methylated sites (Fig. 2A). We identified that 194, 770 CpG sites were hypermethylated in CD3+ subsets than in CD3– subset; 150 sites were hypermethylated in CD3+/CD8+ subset, a higher level than that of CD3+/CD8− subset and that in the CD3−/CD8− subset. In the CD3+/CD8− subset compared to the CD3+/CD8+ or CD3−/CD8− subset, 34 sites were hypermethylated. For 19 and 13 probes that showed Δβ-values > 0.78 in CD3+/CD8+ cells vs. CD3−/CD8− cells and in CD3+/CD8− cells vs. CD3−/CD8− cells, respectively, we could not find any probe that displayed a significant difference in β-values between CD3+/CD8− cells and CD3+/CD8+ cells. Thus, from the 19 and 13 probes, we identified probes that fulfilled the 2nd and 3rd selection criteria, the availability of designable primers and probes for the MethyLight assay, and the correlation between methylation level of the marker and the proportion of CD3+/CD8+ cells or CD3+/CD8− cells. Through this approach, three DNA methylation markers (*NCOR2*, *PARK2*, and *ZSCAN12*) were obtained from the 19 probes, and one DNA methylation marker (*NCOR2*) was obtained from the 13 probes. However, we did not obtain DNA methylation markers that were able to differentiate between the CD3+/CD8+ subset and the CD3+/CD8− subset. A MethyLight assay was performed to identify whether methylation levels assessed by the MethyLight assay correlated with the proportion of CD3+/CD8+ cells in genomic DNA samples, and *NCOR2*, *PARK2*, and *ZSCAN12* exhibited such correlations (Fig. 2B) and no methylation in GC cell lines as a negative control (Supplementary Fig. 1).

### Correlation of *NCOR2*, *PARK2*, and *ZSCAN12* methylation levels with CD3 or CD8 TIL densities in gastric cancer tissue samples

**Figure 3:**
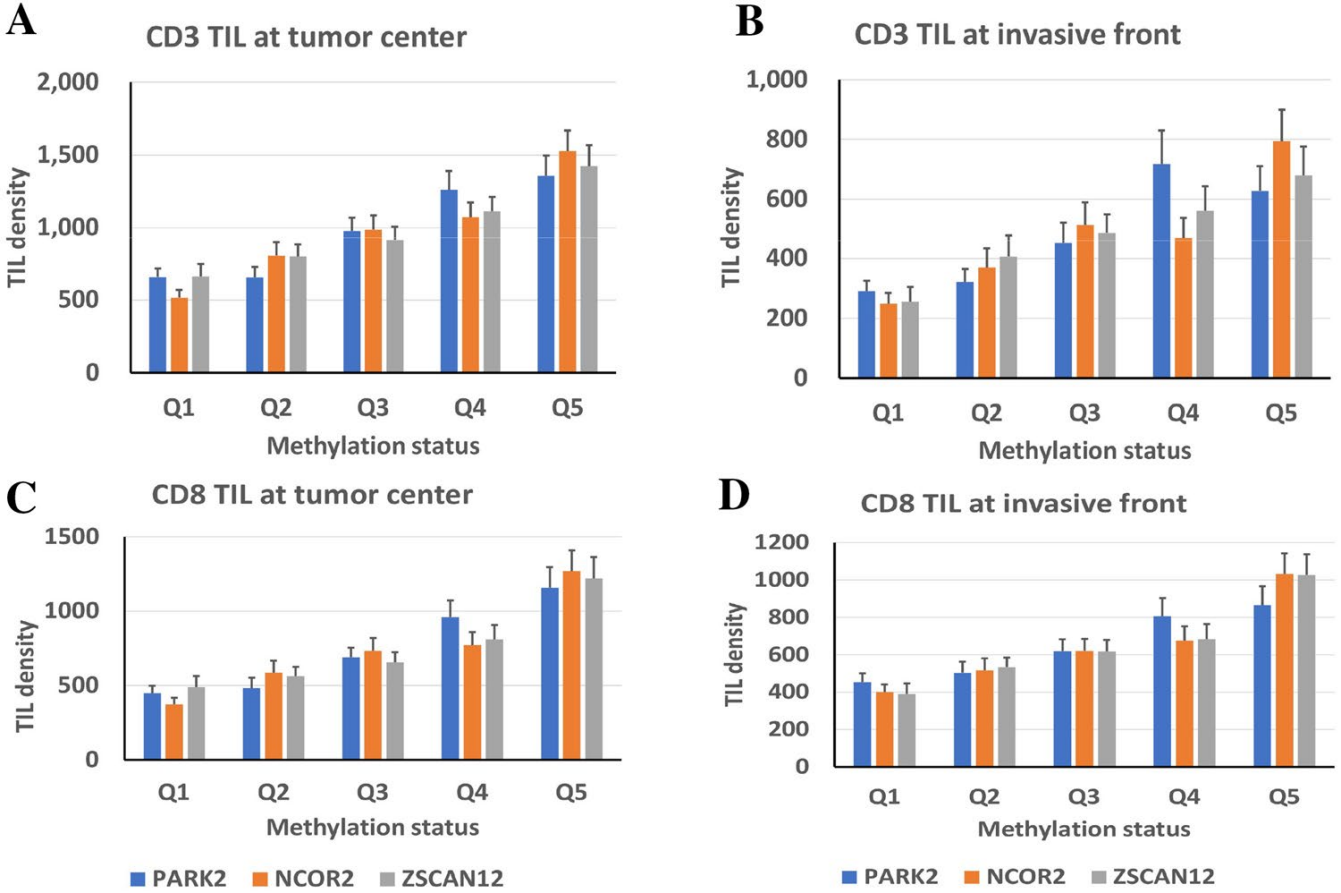
CD3 TIL density (**A**,**B**) or CD8 TIL density (**C**,**D**) at the tumor center (**A**,**C**) or invasive front (**B**,**D**) according to quintile subsets of *NCOR2*, *PARK2*, and *ZSCAN12*. Spearman’s rho and *P*-value are summarized in Supplementary Table 1.

**Figure 4:**
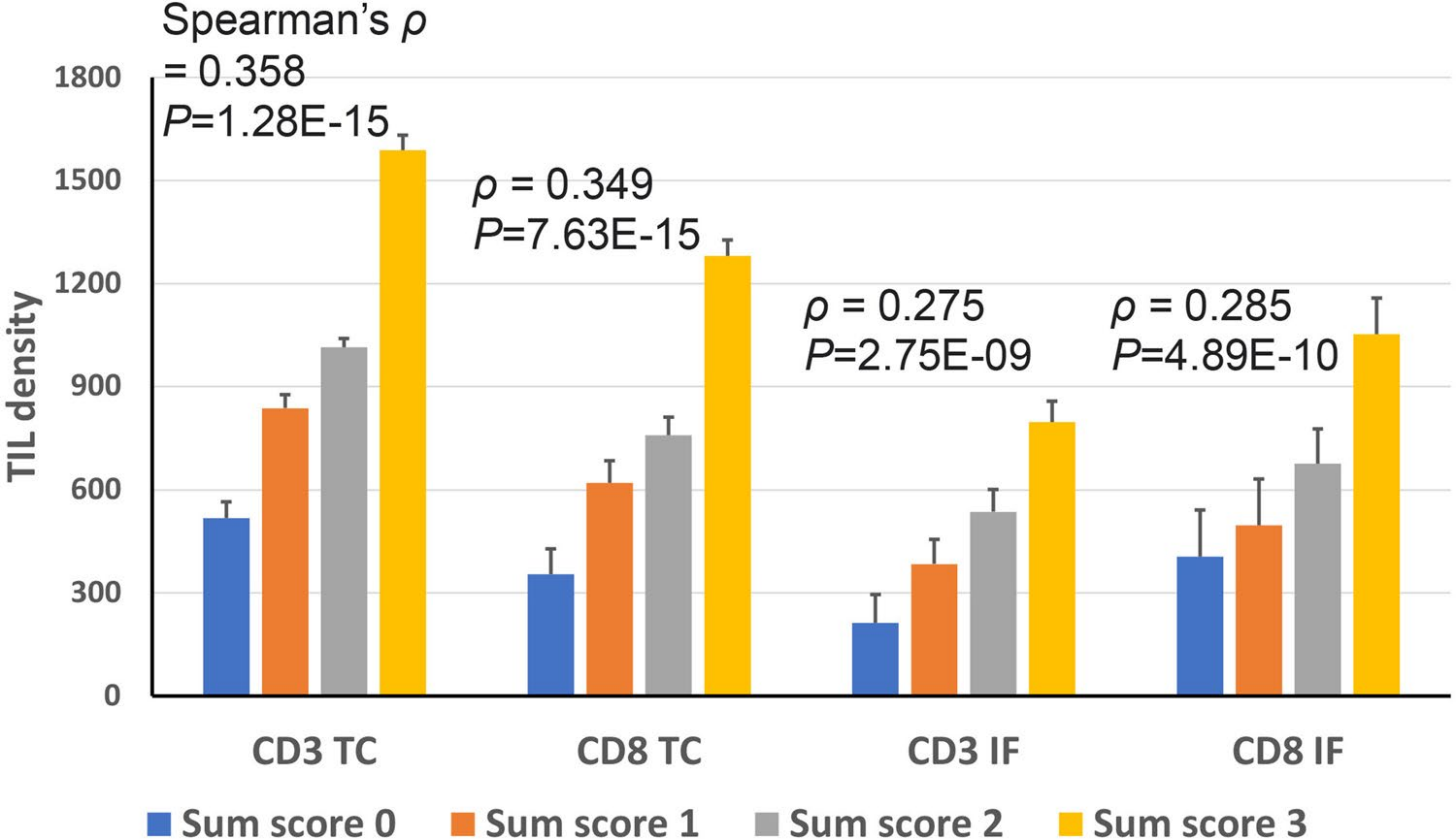
CD3 TIL density or CD8 TIL density at the tumor center (TC) or invasive front (IF) according to the sum score of three DNA methylation markers (*NCOR2*, *PARK2*, and *ZSCAN12*). Spearman test was performed to determine the correlation between TIL density and the sum score.

For a total of 471 cases of advanced GC, *NCOR2*, *PARK2*, and *ZSCAN12* methylation statuses were determined in DNA samples obtained from tissue slides with representative tumor histology. Tissue microarrays were constructed with one tissue core from the tumor center and two tissue cores from the invasive front and subjected to anti-CD3 and anti-CD8 immunohistochemistry. When the densities of CD3+ cells or CD8+ cells were compared among quintile groups of GCs according to the methylation level of each marker, a positive correlation was identified between methylation level and the density of CD3+ or CD8+ cells in the tumor center or invasive front (Fig. 3). With a cutoff set at the median methylation level of each methylation marker, GCs with low methylation and high methylation statuses were scored as 0 and 1, respectively. The sum scores of the three methylation markers showed a correlation with CD3-positive or CD8-positive cell densities at the tumor center or invasive front (Fig. 4 and Supplementary Fig. 2).

### *NCOR2*,* PARK2*, and *ZSCAN12* methylation statuses and their association with the survival of patients with gastric carcinoma

**Figure 5:**
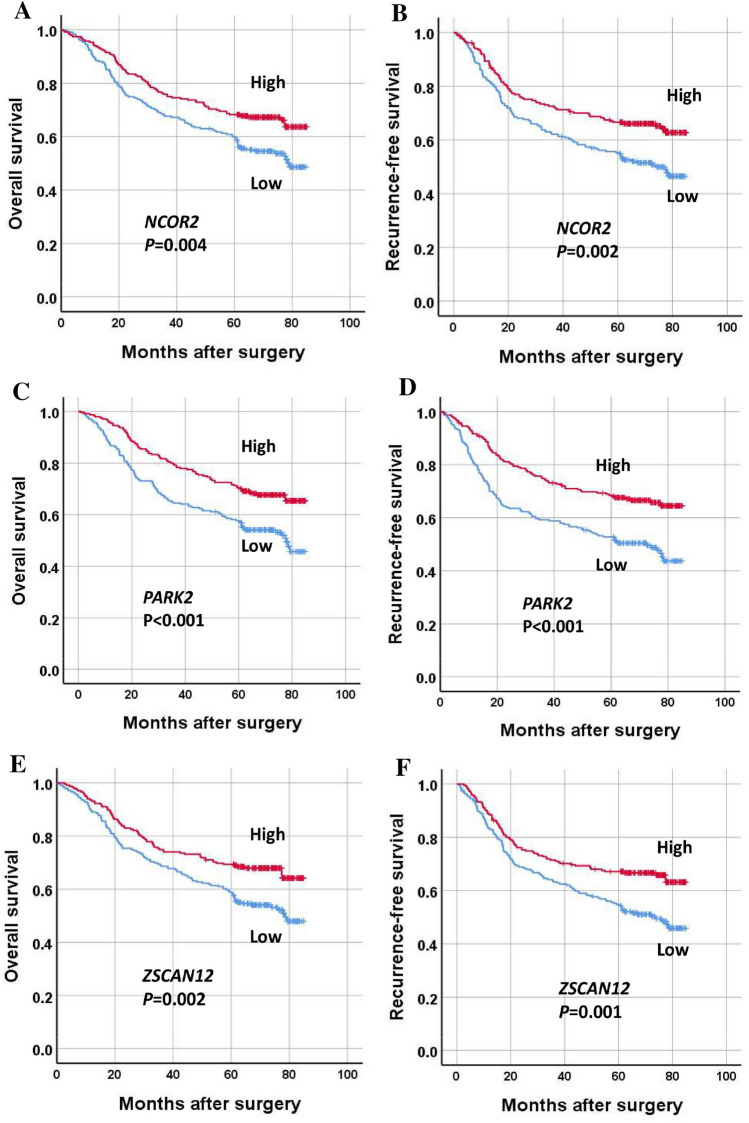
Survival of gastric cancer patients according to methylation status of three DNA methylation markers (*NCOR2*, *PARK2*, *ZSCAN12*). Low and high methylation statuses indicate below and above the median values, respectively. Overall survival (**A**,**C**,**E**) and recurrence-free survival (**B**,**D**,**F**).

**Figure 6:**
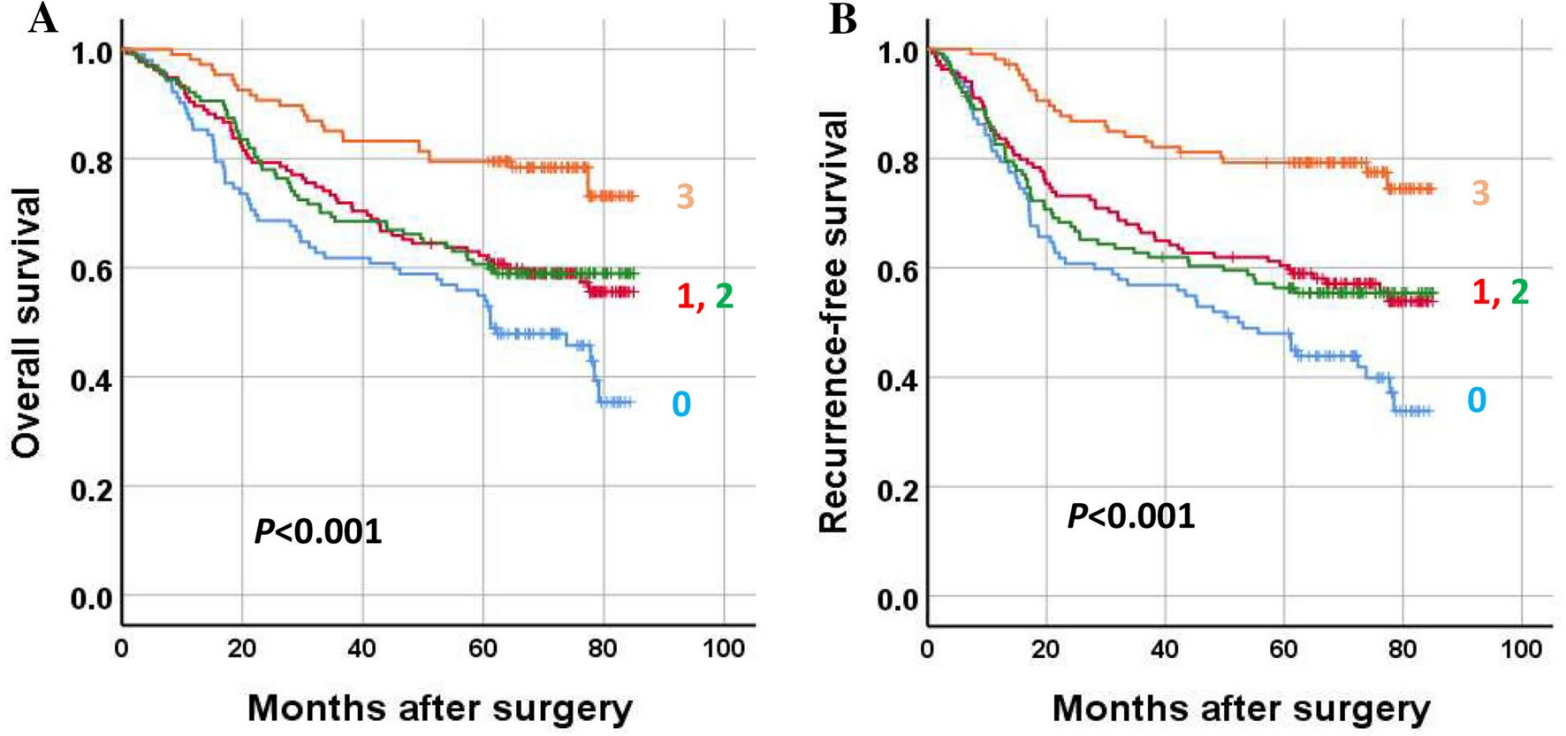
Survival of gastric cancer patients according to the sum score of three DNA methylation markers (*NCOR2*, *PARK2*, *ZSCAN12*). (**A**) Overall survival and (**B**) recurrence-free survival.

When survival analysis was performed, high methylation of each marker was associated with better overall survival and recurrence-free survival in patients with GC (Fig. 5). To identify whether the methylation statuses of individual markers are independent prognostic markers, multivariate analyses were performed with the inclusion of clinicopathological parameters that were found to be significant prognostic factors in univariate analysis. A high methylation status of each marker was found to be marginally significant in multivariate analysis (Table 1). When GCs were sorted into four groups according to the sum score of three methylation markers, GCs with the highest sum (score = 3) showed the best OS and RFS, whereas GCs with the lowest sum (score = 0) exhibited the worst OS and RFS (Fig. 6). On multivariate analysis, the methylation score sum was found to be an independent prognostic parameter for both OS and RFS (Table 1). The methylation score showed close associations with tumor location within the stomach, lymphatic emboli, and N category (Table 2).

**Table 1:** Multivariate analysis of overall survival and recurrence-free survival.

Variables	Overall survival		Recurrence-free survival	
	HR (95% CI)	*P value*	HR (95% CI)	*P* value
***NCOR2* methylation**				
Low (n = 235)	–			
High (n = 236)	0.870 (0.644–1.175)	0.363	0.816 (0.609–1.093)	0.173
***PARK2* methylation**				
Low (n = 234)	–			
High (n = 237)	0.729 (0.539–0.984)	0.039	0.689 (0.513–0.925)	0.013
***ZSCAN12* methylation**				
Low (n = 236)	–			
High (n = 235)	0.728 (0.540–0.983)	0.038	0.695 (0.519–0.932)	0.015
**Sum of methylation scores**		0.017		< 0.001
0 (n = 102)	–			
1 (n = 135)	0.832 (0.568–1.218)	0.344	0.754 (0.522–1.089)	0.132
2 (n = 127)	0.974 (0.658–1.442)	0.895	0.965 (0.663–1.404)	0.852
3 (n = 107)	0.474 (0.288–0.778)	0.003	0.378 (0.231–0.617)	< 0.001

Adjusted for age, tumor subsite, T category, N category, M category, lymphatic emboli, venous invasion, and perineural invasion.

*HR* hazard ratio, *CI* confidence interval.

**Table 2:** Association between methylation score and clinicopathological features.

	Methylation score			*P*-value
	0 (n = 102)	1, 2 (n = 262)	3 (n = 107)	
**Age**				
< 62 years	45 (44.1)	136 (51.9)	56 (52.3)	0.366
≥ 62 years	57 (55.9)	126 (48.1)	51 (47.7)	
**Sex**				
Male	70 (68.6)	174 (66.4)	73 (68.2)	0.897
Female	32 (31.4)	88 (33.6)	34 (31.8)	
**Subsite**				
Involving the cardia	23 (22.5)	68 (26.0)	42 (39.3)	0.013
Not involving	79 (77.5)	194 (74.0)	65 (60.7)	
**Lymphatic emboli**				
Absent	29 (28.4)	90 (34.4)	57 (53.3)	< 0.001
Present	73 (71.6)	172 (65.6)	50 (46.7)	
**Venous invasion**				
Absent	74 (72.5)	192 (73.3)	88 (82.2)	0.154
Present	28 (27.5)	70 (26.7)	19 (17.8)	
**Perineural invasion**				
Absent	47 (46.1)	117 (44.7)	47 (43.9)	0.103
Present	55 (53.9)	145 (55.3)	60 (56.1)	
**T category**				
T2	17 (16.7)	61 (23.3)	31 (29.0)	0.108
T3–T4	85 (83.3)	201 (76.7)	76 (71.0)	
**N category**				
N0	19 (18.6)	84 (32.1)	42 (39.3)	0.004
N1–N3	83 (81.4)	178 (67.9)	65 (60.7)	
**M category**				
M0	87 (85.3)	229 (87.4)	100 (93.5)	0.145
M1	15 (14.7)	33 (12.6)	7 (6.5)	

Values are presented as number (%).

## Discussion

The prognostic impact of TIL assessment in GC was first demonstrated by Davessar et al.’s study, in which the degree of inflammatory response within tumor areas was directly proportional to the longevity of patients with GC, though the study did not differentiate lymphocytes from neutrophils and monocytes in the inflammatory response^11^. However, studies in which lymphocytes or lymphoplasma cells were graded only in the inflammatory response did not show that the degree of TIL is an independent prognostic parameter^12,13^. Lee et al. performed immunohistochemistry for CD3, CD8, and CD45RO and counted immunostained cells using ImageJ software, reporting that high densities of CD3+, CD8+, or CD45RO+ cells are independent prognostic factors in patients with GC^14^. For the subsequent 10 years, more than 20 research papers have been published regarding TIL density and GC patient survival. Meta-analysis results indicate that high densities of CD3+ or CD8+ cells may serve as prognostic biomarkers in patients with GC^15,16^. Although a dozen studies have indicated CD3 or CD8 TIL density as a potential prognostic biomarker, these studies varied in the methodology utilized for the evaluation of CD3 or CD8 TIL as well as cutoff values for binary division. To include TIL assessment in routine pathological reporting, standardized methodology for TIL assessment is required.

CpG sites that exhibit differential methylation between cell types can be applied to estimate the fraction of specific cell types in tissues. Such an attempt to assess the fraction of leukocyte infiltration into a tumor based on differential methylation statuses on CpG sites has been demonstrated by Carter et al., who estimated the fraction of leukocytes in ovarian cancer tissues based on methylation signatures generated by 200 CpG sites^17^. However, the utilization of the methodology to assess leukocyte fraction was limited to ovarian cancer tissues. In addition, Aran et al. developed a computational method, leukocyte unmethylation for purity (LUMP), which uses the average methylation levels of 44 CpG sites to estimate the leukocyte fraction. LUMP has been applied to human cancers regardless of tissue type^18^. However, those methodologies cannot provide information regarding the densities of different cell types of TILs and only provide information about the overall leukocyte fraction. According to a recent study by Chakravarthy et al., their computational methodology provides estimates for different infiltrating cell populations as a fraction of the overall sample^19^. Overall, the above three computational methodologies are dependent on Illumina Infinium technology-mediated assessment of the methylation level of dozens of CpG sites. In contrast, the Fuks team developed a pyrosequencing-based assessment of five methylation markers, which enabled recapitulation of TIL density in breast cancer^10^. The five markers comprise single CpG sites of five individual genes, including *INA*, *KLHL6*, *PTPRCAP*, *RASSF1*, and *SEMA3B*, which are different from the three methylation markers in the present study^10^. During the submission of our manuscript, it was reported that Zou et al. developed DNA methylation signature for CD8+ TILs that could assess CD8+ TILs in colorectal cancers, using three individual CpG sites that are located in the low-CpG density regions and highly differentially methylated between CD8+ T cells and other cells^20^. The selected CpG sites are not located within CpG island loci, so that classical MethyLight assay could not assess methylation levels of these single CpG sites. Zou et al. utilized a modified-MethyLight assay, so-called an assay for the quantitative analysis of single base methylation which showed high correlation compared with pyrosequencing^21^. The DNA methylation signature was demonstrated to predict prognosis for patients with colorectal cancer.

In the present study, genome-wide methylation profiling was applied to identify methylation markers that signify TILs in GCs and are useful for the prediction of prognosis in GC. For three methylation markers, *NCOR2*, *PARK2*, and *ZSCAN12*, correlation of their methylation levels with the densities of CD3+ or CD8+ cells at the tumor center or invasive front was found (Fig. 3). The sum of the methylation scores of the three methylation markers also exhibited a correlation with CD3+ or CD8+ cell densities at the tumor center or invasive front (Fig. 4). In both univariate and multivariate survival analyses, the sum of the methylation scores of the three methylation markers was associated with GC patient prognosis in terms of both OS and RFS (Fig. 6 and Table 1). In the present study, we used the MethyLight technology, real-time methylation-specific PCR, which provides semiquantitative information regarding the fraction of heavily methylated DNA alleles in DNA samples. The MethyLight assay is powerful for detecting a low fraction of methylated DNA alleles among a high fraction of unmethylated DNA alleles^22^. The pyrosequencing methylation assay may be an alternative solution for assessing methylation levels at three genes. Nonetheless, a single batch of the MethyLight assay with a real-time PCR machine with a 384-well plate enables rapid assessment of the methylation status of three methylation markers using more than 100 different tissue samples. In the present study, we did not analyze whether the sum of the methylation scores of the three methylation markers can be applied to other tissue types of human cancers for the recapitulation of TIL density. Further study is required to validate its usefulness in the evaluation of TILs in other cancer tissue types.

To assess the CD8+ TIL density by evaluating the immunostained slides using an image analyzer and software requires dedicated equipment, which is an obstacle for TIL evaluation to be applied to routine practice of pathology. The methylation score of the present study does not require special equipment, so it will contribute to the introduction of TIL evaluation into routine practice of pathology. In addition, the methylation score is likely to enable more precise prediction of the prognosis by stratifying patients with microsatellite-unstable or -stable GC (Supplementary Fig. 3) and serve as a biomarker to find patients who are indicated for immunotherapy.

In summary, a MethyLight-based assessment of three markers (*NCOR2*, *PARK2*, and *ZSCAN12*) was developed and found to recapitulate TIL densities in GC. Methylation scores of three markers were closely associated with TIL densities and served as an independent prognostic marker in patients with GC. Further studies are required to validate the usefulness of MethyLight-based assessment of three methylation markers as an alternative tool for TILs assessment.

## Material and methods

### Tissue samples and clinicopathological analysis

Fresh tumor tissues were obtained from three patients who underwent curative surgery for advanced gastric carcinoma (AGC) at Seoul National University Hospital, Seoul, Korea. Formalin-fixed, paraffin-embedded (FFPE) tumor material from a consecutive series of AGC cases (n = 471) was retrieved from the surgical files of the Department of Pathology, Seoul National University Hospital, Seoul, Korea. The inclusion and exclusion criteria for retrospective patient selection were described previously^23^. The patients underwent gastrectomy and D2 lymph node dissection for AGC between January 2007 and December 2008. Demographic and baseline characteristics are summarized in Supplementary Table 2. The ages of the patients ranged from 23 to 86 years, with an average of 60.5 years, and the male to female ratio was 2.06:1. Clinical and histological data were retrieved from the electronic medical records, including tumor subsites within the stomach, lymphatic emboli, venous invasion, perineural invasion, and tumor-node-metastasis stage (American Joint Committee on Cancer, 7th edition). This study was approved by the Institutional Review Board of Seoul National University Hospital (approval no. C-1803-099-931) which waived the requirement to obtain informed consent, and was conducted in accordance with the Declaration of Helsinki.

### Genome-wide methylation analysis of sorted cells

Three fresh tumor tissues were enzymatically and mechanically dissociated into cell suspensions to which an APC-tagged anti-CD3 antibody and a PE-tagged anti-CD8 antibody (BD Biosciences, San Jose, CA, USA) were added. Fluorescently tagged-cell suspensions were subjected to fluorescence-activated cell sorting, and separated cells, including CD3–/CD8–, CD3+/CD8–, and CD3+/CD8+ cells, were collected. Genomic DNA was extracted using a QIAamp DNA Mini Kit (QIAGEN, Hilden, Germany) and then modified with an EZ DNA methylation kit (Zymo Research, Irvine, CA, USA). Differences in DNA methylation between sorted cells were determined by MethylationEPIC BeadChip analysis (Illumina, San Diego, CA, USA). Array CpG probes that have detection p-value ≥ 0.05 in over 25% samples were filtered out. And then filtered data was subjected to background correction & dye bias equalization by R methylumi & lumi package. To reduce Infinium I and Infinium II assay bias, corrected signal value was normalized by BMIQ (Beta Mixture Quantile) method.

### Selection of DNA methylation markers that show correlation between their methylation levels and DNA concentrations from tumor-infiltrating lymphocytes

We sought to develop DNA methylation markers that are specific for two subsets, CD3+/CD8+ and CD3+/CD8− cells. Candidate MethylationEPIC probes were selected on the criteria: (1) Δβ-value > 0.78 in CD3+/CD8+ cells vs. CD3−/CD8− cells or in CD3+/CD8− cells vs. CD3−/CD8− cells, (2) availability of designable primers and probes for MethyLight assay, and (3) correlation between methylation levels of the gene and proportion of CD3+/CD8+ cells or CD3+/CD8− cells.

### Semiquantitative assessment of DNA methylation markers in gastric carcinomas

Through microscopic examination of all available tissue slides, areas up to 1 cm^2^ where tumor cells were the most abundant and representing the most prevalent histology of the individual case were marked. The corresponding areas on unstained-tissue slides were scraped with knife blades, and samples were collected into microtubes containing tissue lysis buffer and proteinase K. The microtubes were kept at 55 °C for 24 h, incubated at 95 °C for 30 min, and centrifuged. The supernatants were transferred to new microtubes. Bisulfite modification of the supernatants was conducted using an EZ DNA methylation kit (Zymo Research). For assessment of methylation levels at the three DNA methylation markers, the modified DNA samples were subjected to MethyLight analysis, which was performed as described previously^24^. The oligonucleotide sequences of the primers and probes used for the three DNA methylation markers are listed in Supplementary Table 3.

### Cell culture and reagents

Four human GC cell lines (NUGC-2, SNU-16, SNU-620, and SNU638), obtained from the Korean Cell Line Bank (Seoul, South Korea) were grown in RPMI-1640 medium supplemented with 10% fetal bovine serum and 1% antibiotic solution containing penicillin and streptomycin. Cells were incubated at 37ºC in a humidified atmosphere of 5% CO_2_. Genomic DNA was extracted from these cell lines using a QIAamp DNA Mini Kit (QIAGEN).

### Tissue microarray and immunohistochemical staining for CD3 and CD8

After microscopic examination, an area of 2 mm in diameter was marked on the tumor center, and two areas were marked on the invasive front. Tissue blocks were available for 453 cases, and the marked areas of the corresponding tissue blocks were punched to construct a tissue microarray (TMA). Four-micrometer-thick sections from TMA tissue blocks were stained with an anti-CD3 antibody (1:200, Dako, Glostrup, Denmark) and anti-CD8 antibody (1:100, Neomarkers, Fremont, CA, USA). CD3-positive or CD8-positive cells were counted using the open-source software QuPath^25^. The output was the CD3 TIL density and CD8 TIL density (number of cells per mm^2^ of tissue) of each core.

### Statistical analysis

Statistical analysis was performed with SPSS software for Windows, version 25.0 (SPSS, Chicago, IL, USA). To determine whether CD3 and CD8 TIL densities were normally distributed, a normality test was performed using Shapiro–Wilk’s W test. *P* values less than 0.001 were considered significant, rejecting the null hypothesis that the data were normally distributed. Spearman test was used to determine the correlation between variables. Recurrence-free survival (RFS) was measured from the date of surgery to the date of recurrence or the date of death from any cause (whichever came first). The Kaplan–Meier log rank test was conducted to compare overall survival (OS) and RFS times across groups. A Cox proportional hazards model was used to calculate the hazard ratio (HR), and we included in the multivariate analysis all covariates with a *P value* < 0.05 from univariate analysis to adjust for potential confounders: age (younger and older), tumor subsite (involving and not involving cardia), T category, N category, M category, lymphatic emboli, venous invasion, perineural invasion, CD3 TIL density at the tumor center, CD3 TIL density at the invasive front, CD8 TIL density at the tumor center and CD8 TIL density at the tumor center. Backward stepwise elimination was carried out with a *P value* = 0.05 as a threshold to select variables for the final model. The final results are expressed as HRs with 95% confidence intervals (CIs).

### Ethics approval

This study was approved by the Institutional Review Board of Seoul National University Hospital which waived the requirement to obtain informed consent because of the retrospective nature of this study.

## Supplementary Information

Supplementary Information.

## Data Availability

The datasets generated during and/or analysed during the current study are available from the corresponding author on reasonable request.
