# Supplementary material for: Methylation statuses of NCOR2, PARK2, and ZSCAN12 signify densities of tumor-infiltrating lymphocytes in gastric carcinoma
Source: Sci Rep. 2022 Jan 17;12:862. doi: 10.1038/s41598-022-04797-9 (PMC8763922; doi:10.1038/s41598-022-04797-9)

Methylation statuses of NCOR2, PARK2, and ZSCAN12 signify densities of tumor-infiltrating lymphocytes in gastric carcinoma

Xianyu Wen,[1,2,*] Hye-Yeong Jin,[1,2,*] Meihui Li,[1,2] Younghoon Kim,[1,2] Nam-Yun Cho,[2] Yoonjin Kwak,[1] Jeong Mo Bae,[1,2] Hye Seung Lee,[1] Gyeong Hoon Kang[1,2]

[1]Department of Pathology, Seoul National University College of Medicine, Seoul, Korea

[2]Laboratory of Epigenetics, Cancer Research Institute, Seoul National University College of Medicine, Seoul, Korea

*, These two authors contributed equally to this article.

Supplementary Table 1. Supplementary Table. Summary of Spearman's rho and P-value in correlation tests between quintile subsets of *NCOR2*, *PARK2*, and *ZSCAN12* methylation and CD3 TIL density or CD8 TIL density at the tumor center or invasive front

		Quintile subsets		
		NCOR2 methylation	PARK2 methylation	ZSCAN12 methylation
Tumor center	CD3 TIL density	0.340<br>3.52E-14	0.268,<br>3.39E-09	0.277<br>1.04E-09
	CD8 TIL density	0.332<br>1.72E-13	0.293<br>1.09E-10	0.274<br>1.77E-09
Invasive front	CD3 TIL density	0.227<br>1.07E-06	0.159<br>6.92E-04	0.226<br>1.16E-06
	CD8 TIL density	0.250<br>5.27E-08	0.150<br>1.25E-03	0.279<br>1.10E-09

Supplementary Table 2. Demographic and clinicopathologic features of the gastric cancer patients.

Characteristic		No. of patients (n=471)
Age, year	Median (range)	61 (23–86)
Sex	Male	317 (67.3%)
	Female	154 (32.7%)
Subsite	Involving the cardia	133 (28.2%)
	Not involving the cardia	338 (71.8%)
Lymphatic emboli	Absent	176 (37.4%)
	Present	295 (62.6%)
Venous invasion	Absent	354 (75.2%)
	Present	117 (24.8%)
Perineural invasion	Absent	211 (44.8%)
	Present	260 (55.2%)
T category	T2a	109 (23.1%)
	T2b	177 (37.6%)
	T3	165 (35.0%)
	T4	20 (4.2%)
N category	N0	145 (30.8%)
	N1	87 (18.5%)
	N2	90 (19.1%)
	N3a	95 (20.2%)
	N3b	54 (11.5%)
M category	M0	416 (88.3%)
	M1	55 (11.7%)

Supplementary Table 3. Oligonucleotide sequences for the three DNA methylation markers

Gene		Oligonucleotide sequences
NCOR2	forward	GATCGTTGGAGGTTCGACGTT
	reverse	AACCCACCGACGACAAATACG
	probe	6FAM-CCGCCTAACCCCACGACTAAACCGTAAA-BHQ1
PARK2	forward	GGATGGAACGGTTGCGATAGATT
	reverse	AACGAATACTATCCTAACACTAACTCAAC
	probe	6FAM-ACAACGCAAAACCGAACCCGAACGAC-BHQ1
ZSCAN12	forward	CGCGTTTCGGTTTGGGAGTA
	reverse	CTCCTCTCTATAATTACCCGACCG
	probe	6FAM-CGCCCCAACTAAACAACGCAAATTCCAACT-BHQ1

Supplementary Figure 1. MethyLight assay of three methylation markers (NCOR2, PARK2, and ZSCAN12) in gastric cancer cell lines (NUGC-2, SNU-16, SNU-620, and SNU-638) as a negative control and CD8-positive T cells (from two gastric cancer patients) as a positive control

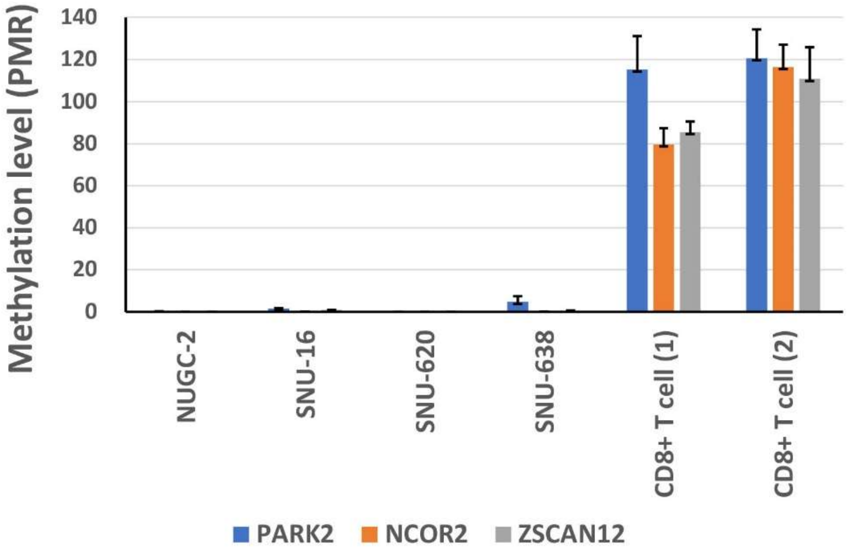

Supplementary Figure 2. Representative photomicrographs demonstrating histologic (A, B, and C) and CD8 immunohistochemistry features (D, E, and F) of gastric cancers (GCs). A GC with methylation sum score of 0 (A & D); a GC with methylation sum score of 1 (B & E); a GC with methylation sum score of 3 (C & F)

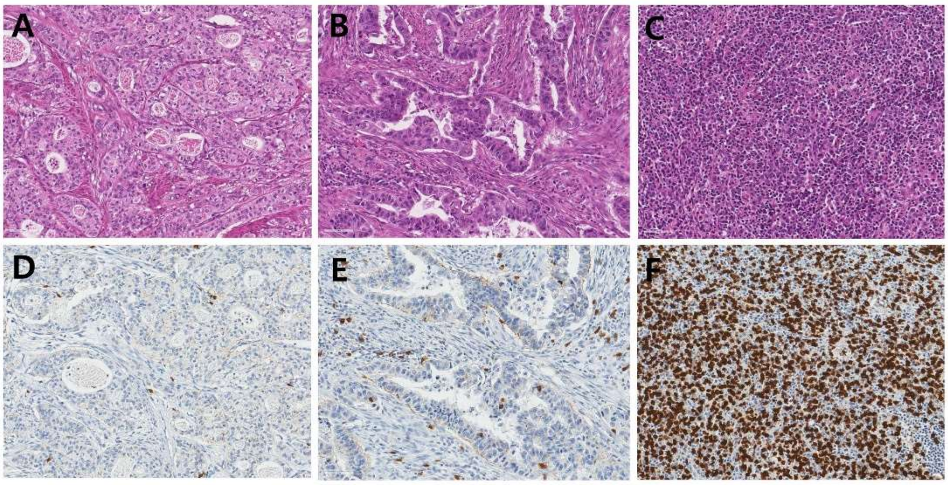

Supplementary Figure 3. Kaplan-Meier survival analysis of overall survival (A) and recurrence-free survival (B) with respect to the combinatory statuses of microsatellite instability (MSI) and methylation sum score. Gastric cancer cases with methylation sum score of 0-2 were classified into methylation sum score (MeSc)-low, which gastric cancer cases with methylation sum score of 3 were classified into MeSc-high.

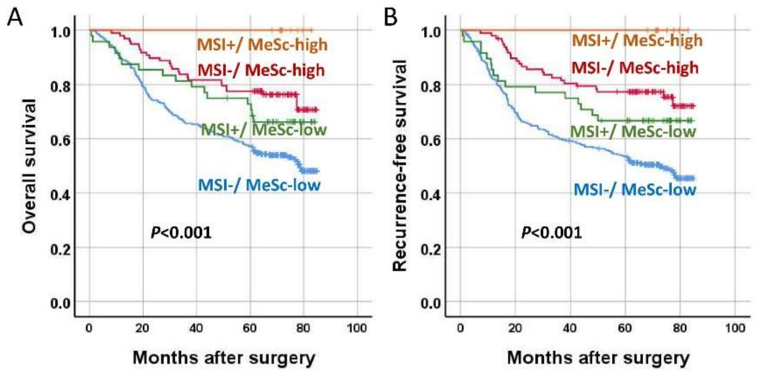

Supplement: Supplementary file 1 — Supplementary Information. [file 41598_2022_4797_MOESM1_ESM.pdf]
